# Fabrication of avidin-stabilized gold nanoclusters with dual emissions and their application in biosensing

**DOI:** 10.1186/s12951-022-01512-8

**Published:** 2022-06-27

**Authors:** Zhenrong Tang, Fengjiao Chen, Dan Wang, Dongmei Xiong, Shaoying Yan, Shengchun Liu, Hua Tang

**Affiliations:** 1 Department of Endocrine and Breast Surgery, The First Affiliated Hospital of Chongqing Medical University, Chongqing, 400042 China; 2 Guangshan County People’s Hospital, Xinyang, 465450 Henan China; 3 Key Laboratory of Molecular Biology for Infectious Diseases (Ministry of Education), Department of Infectious Diseases, Institute for Viral Hepatitis, The Second Affiliated Hospital, Chongqing Medical University, 1 Yi Xue Yuan Road, Chongqing, 400016 China; 4 Nursing School of Chongqing Medical and Pharmaceutical College, Chongqing, 401331 China; 5 Department of Clinical Laboratory, The First Affiliated Hospital of Nanchang University, Nanchang, 330006 Jiangxi China

**Keywords:** Avidin-stabilized gold nanoclusters (Av–Au NCs), Protein-stabilized gold nanoclusters (Prot-Au NCs), Dual-functional, Biosensor, Detect

## Abstract

Protein-stabilized gold nanoclusters (Prot-Au NCs) have been widely used in biosensing and cell imaging owing to their excellent optical properties and low biotoxicity. However, several Prot-Au NCs reported in the literature do not retain the biological role of the protein, which greatly limits their ability to directly detect biomarkers. This study demonstrated for the first time the successful synthesis of dual-function avidin-stabilized gold nanoclusters (Av–Au NCs) using a one-pot method. The resulting Av–Au NCs exhibited intense blue and red emissions under 374 nm excitation. Furthermore, the Av–Au NCs retained the native functionality of avidin to bind to biotin. When DNA strands modified with biotin at both ends (i.e., linker chains) were mixed with Av–Au NCs, large polymers were formed, indicating that Av–Au NCs could achieve fluorescence signal amplification by interacting with biotin. Taking advantage of the aforementioned properties, we constructed a novel enzyme-free fluorescent biosensor based on the Av–Au NCs-biotin system to detect DNA. The designed fluorescent biosensor could detect target DNA down to 0.043 nM, with a wide line range from 0.2 nM to 20 µM. Thus, these dual-functional Av–Au NCs were shown to be an excellent fluorescent material for biosensing.

**Graphic abstract:**

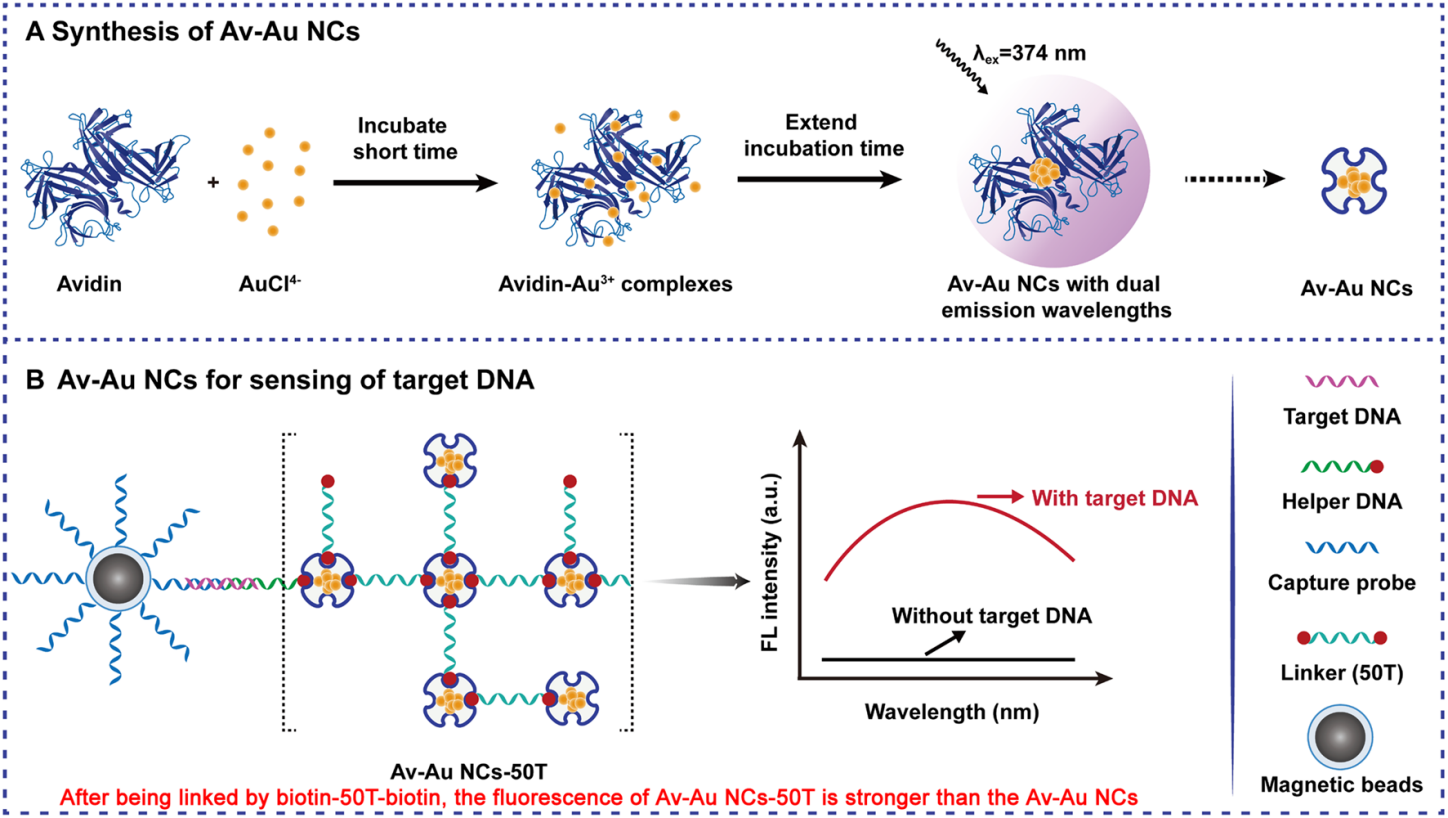

**Supplementary Information:**

The online version contains supplementary material available at 10.1186/s12951-022-01512-8.

## Highlights

1. Avidin-stabilized gold nanoclusters (Av–Au NCs) were synthesized for the first time by a water-bath method.
2. The synthesized Av–Au NCs not only exhibited intense blue and red emissions under 374 nm excitation, but also retained the native functionality of avidin to bind to biotin.
3. The fluorescent signal amplification system constructed by the interaction of Av–Au NCs with biotin was successfully applied to detect target DNA in vitro.

**Supplementary Information:**

The online version contains supplementary material available at 10.1186/s12951-022-01512-8.

## Background

Recently, gold nanoclusters (Au NCs) have attracted increasing attention in the fields of biosensing, theranostics, and bioimaging owing to their unique physicochemical properties [1–6]. Au NCs are nanostructures composed of cores of several to tens of gold atoms and extra-nuclear ligands, generally approximately 1–2 nm in size. When the size of a nanostructure is comparable to the Fermi wavelength of the conduction electrons, the electron energy level becomes discontinuous owing to the quantum size effect, which may give rise to size-dependent luminescence, resulting in properties that are much different from those of classical metallic nanoparticles [7]. Compared to traditional organic fluorophores and quantum dots, Au NCs are not easily photobleached and have low toxicity, so they have great potential for application in biosensing [8, 9]. In the synthesis and development of Au NCs, ligands that encapsulate the gold core have gradually evolved from small molecules of sulfhydryl compounds [10] to large-molecule polymers [11], dendrimers [12], DNA [13], peptides [14], and proteins [15]. Au NCs stabilized by proteins are ideal candidate materials for biological applications owing to their high biocompatibility, excellent photoluminescence, good stability across a wide range of pH values, and suitability for biolabeling and targeting [16].

Various natural and synthetic histidine-rich and cysteine-rich proteins or peptides have been employed in the synthesis of Au NCs, including bovine serum albumin (BSA) [15], horseradish peroxidase (HRP) [17], pepsin [18], insulin [19], lysozyme [20], human transferrin [21, 22], papain [23], trypsin [24], DNase 1 [25], β-lactoglobulin [26], and neuropeptide Y [27]. However, only a few reports on bioactive Prot-Au NCs have claimed that the biological role of the protein has not changed. For example, Liu et al*.* [19] reported that they synthesized insulin-stabilized Au NCs and retained the function of insulin, and Guével’s group [21] synthesized transferrin-stabilized Au NCs with the ability to target transferrin receptors. Nevertheless, protein-stabilized Au NCs often interact directly with small biological molecules or heavy metal ions to change the fluorescence intensity of the Au NCs to achieve the purpose of detecting target substances [28, 29]. Biomarkers that do not directly interact with Au NCs cannot be directly detected. Therefore, the goal of this project was to determine a suitable protein to encapsulate Au NCs and retained its biological role so that encapsulated Au NCs could be applied for the construction of fluorescent biosensors.

Among many proteins of vital importance, avidin is of prime interest. Given its strong affinity for biotin molecules, avidin is used as a universal tool of signal amplification or biomolecule extraction in molecular biology research [30, 31]. However, as of yet, there have been no reports on the synthesis of avidin-stabilized gold nanoclusters (Av–Au NCs). Avidin is a basic glycoprotein composed of four identical subunits, each of which contains two cysteine residues, four tryptophan residues, and one tyrosine [32]. The disulfide bond formed by the half-cysteine may be the nucleation site of Au (0) or Au (III) [33]. Avidin remains stable for a long time over a wide range of temperature and pH [32]; thus, the commonly used method to synthesize Au NCs, a 38 ℃ water bath, is likely to have no significant effect on its affinity with biotin. Therefore, the properties of avidin indicate that one-pot synthesis of Av–Au NCs could be possible.

**Scheme 1.**
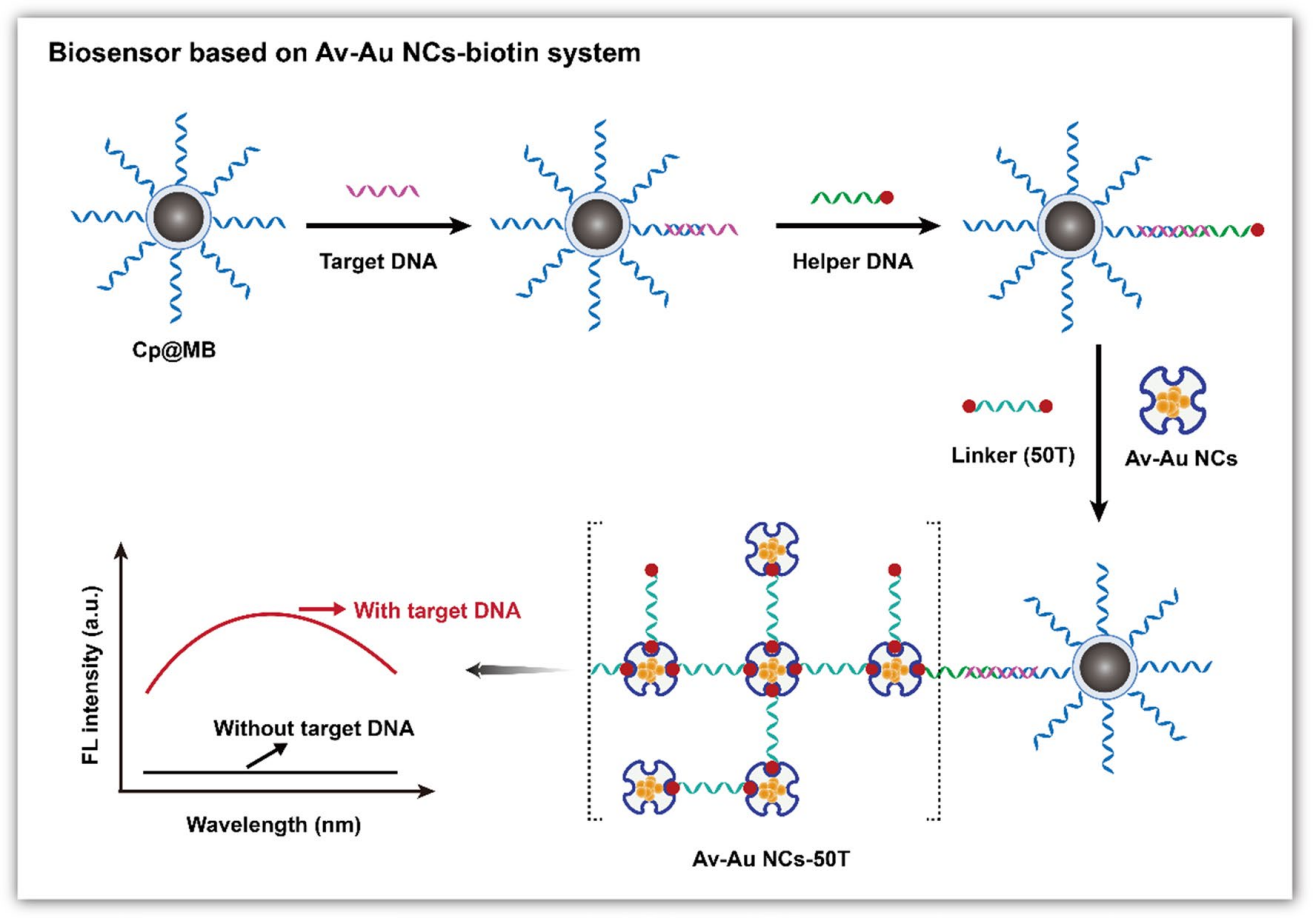
Diagram of the fluorescence biosensor for target DNA detection based on the Av–Au NCs-biotin signal amplification system

This work has two innovative aspects. (I) A one-pot method was used to synthesize Av–Au NCs. During a typical synthesis protocol, a solution of 2 mg/mL avidin was added to an equal volume of 0.4 mM HAuCl_4_, and the pH of the mixed solution was adjusted to 12 to synthesize Av–Au NCs. (II) The synthetic Av–Au NCs retained the native ability of avidin to bind to biotin; thus, Av–Au NCs have the potential to be widely used in the field of biosensing for signal amplification. A random DNA sequence was used as a detection template, and the design of the fluorescent biosensor for detecting target DNA was based on the Av–Au NCs-biotin signal amplification system, shown in Scheme 1. First, magnetic beads (MBs) were synthesized, and capture DNA (Cp) chains were modified on the surface of the MBs with cross-linking agent. Target DNA was then captured by these specific MBs. A DNA sequence with biotin at one end (helper DNA) was then complementary to a second part of the target DNA. Thus, the Cp chains, the helper DNA and target DNA formed a triple-stranded "sandwich" structure through complementary base pairing. Then, the linker chains modified with biotin at both ends (50 T) and Av–Au NCs were added to the above solution. Owing to the affinity between biotin and Av–Au NCs, a large number of Av–Au NCs were cross-linked on the surface of the MBs. As a result, a strong fluorescence signal was obtained, which was positively correlated with the concentration of target DNA. We demonstrated that avidin-functionalized fluorescent Au NCs could be obtained through a simple, one-pot synthetic route and applied for biosensing owing to the bioactivity retained by Av–Au NCs.

## Results and discussion

### Preparation and characterization of Av–Au NCs

**Fig. 1:**
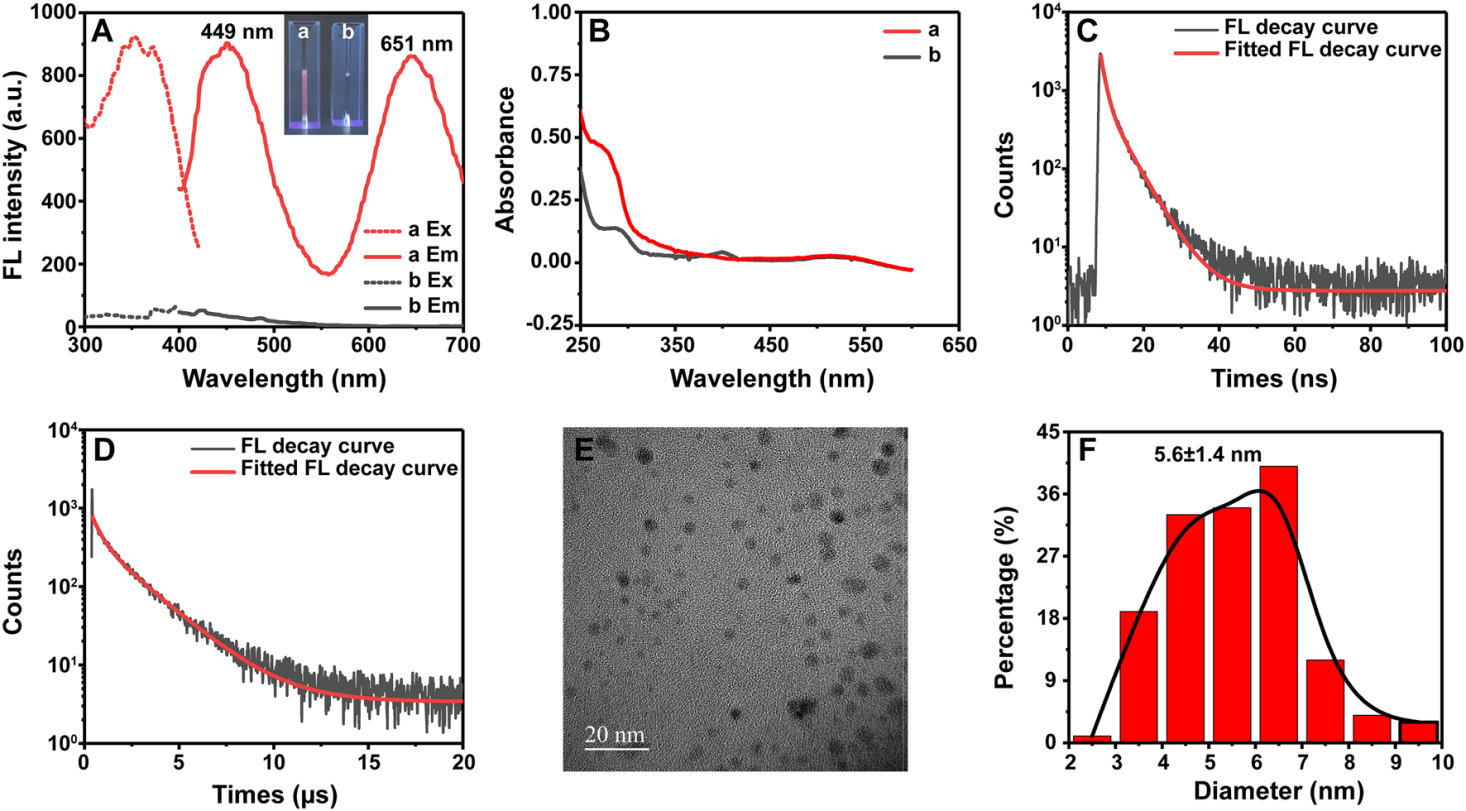
Characterization of Av–Au NCs. **A** Fluorescence spectra of Av–Au NCs (a, red lines) and avidin (b, grey line): dashed and solid lines represent the excitation and emission fluorescence spectra, respectively. Slit widths of excitation: 10 nm, slit widths of emission: 10 nm, and voltage: 700 V. Inset in part (**A**): photographs of the aqueous solutions of Av–Au NCs (a) and avidin (b) taken under 365 nm UV light. **B** Ultraviolet–visible absorption spectrum of Av–Au NCs (a, red lines) and avidin (b, grey line). **C** Fluorescence decay profiles of Av–Au NC solution at 449 nm (excitation at 374 nm). **D** Fluorescence decay profiles of Av–Au NCs solution at 651 nm (excitation at 374 nm). **E** High-resolution transmission electron microscopy image of the Av–Au NCs with 20 nm scale. **F** Size distribution histogram of Av–Au NCs

According to previous literature [20, 33–35], proteins containing cysteine, tryptophan, and tyrosine residues have been used as stabilizers and reducing agents for the synthesis of Au NCs under alkaline pH conditions (pH 12). In the present study, avidin was used to synthesize Av–Au NCs. After adjusting the concentration of avidin and HAuCl_4_, low-concentration avidin-stabilized Au NCs emitted pink fluorescence (inset of Fig. 1A) when excited under a 365 nm UV lamp, indicating the successful synthesis of Av–Au NCs with multi-peak fluorescence. In Fig. 1A, the dashed and solid lines represent the excitation and emission spectra, respectively. The as-prepared Av–Au NCs displayed dual emission wavelengths at ~ 449 nm and ~ 651 nm when excited at 374 nm (Fig. 1A, red lines), and there was no obvious fluorescence in the avidin solution without the addition of HAuCl_4_ (Fig. 1A, gray lines). Several studies have reported that the fluorescence of Au NCs depends on the size of the cores of Au NCs according to the spherical Jellium model [36]. The experimental results demonstrated the synthesis of both large and small Av–Au NCs. The red emission-wavelength of Av–Au NCs was close to the red emission from DNase 1-stabilized Au_25_ NCs (λ_em_ = 640 nm) [25] and that from BSA-mediated Au_25_ NCs (λ_em_ = 640 nm) [15], and the blue emission peak of Av–Au NCs was consistent with that from Lys VI-encapsulated Au_8_ NCs (λ_em_ = 455 nm) [20]. The slight difference in emission wavelength of Au NCs of a similar size was due to different surface ligands [37].

Compared to the same concentration of pure protein (curve b in Fig. 1B), the as-prepared solution exhibited a typical protein absorbance peak and an absence of localized surface plasmon resonance bands (~ 520 nm) [18], suggesting that no gold nanoparticles were formed (curve a in Fig. 1B). The photoluminescence quantum yield (PLQY) of Av–Au NCs in aqueous solution was ~ 19.4%, which was obviously higher than that of previously reported Prot-Au NCs [38]. Chevrier’s group [39] used proteases to degrade the outer shell of BSA-stabilized Au NCs, causing a decrease in fluorescence intensity and quantum yield. Therefore, the high PLQY of Av–Au NCs observed in the current study might be because avidin was relatively resistant to the alkaline synthesis environment [32], which enabled the integrity of avidin to be maintained during the synthesis of Au NCs.

Figure 1C and D show the fluorescence decay curves of Av–Au NCs at 449 nm and 651 nm, respectively. The average lifetime of Av–Au NCs at 449 nm was measured to be 3.5 ns by integrating two components of 1.19 ns (39.31%) and 5.05 ns (60.69%). However, the lifetime of Av–Au NCs at 651 nm was observed to be on the microsecond scale, suggesting that the emission from Av–Au NCs at 651 nm was mainly phosphorescence.

The high-resolution transmission electron microscopy (HRTEM) image clearly showed that the formed Av–Au NCs were evenly distributed (Fig. 1E). Figure 1F showed narrow distribution of 5.6 nm ± 1.4 nm diameter of Av–Au NCs measured from HRTEM, which was similar to that of other Prot-Au NCs reported in previous studies [33, 40, 41]. The above experiments showed that Av–Au NCs were successfully synthesized by a simple biomineralization process.

### XRD and FTIR studies of Av–Au NCs.

Analysis of X-ray diffraction (XRD; Rigaku Smart Lab) patterns was performed to determine the structure of Av–Au NCs. Unfortunately, the simple XRD pattern of the as-prepared lyophilized powder prevents us to obtain detailed structural information of Av–Au NCs. The as-synthesized Av–Au NCs crystallinity and diffracted angles were examined from 10 to 90 degrees (Additional file 1: Fig. S1A). By referring to Au standard card (PDF No. 04–0784), Av–Au NCs has no acicular diffraction peaks of gold-based nanomaterials but only two wide peaks at 17° and 61°. It might be due to the small size [42], and less content [43] of Av–Au NCs, which implies that the Au atoms mainly exist in the form of Au clusters [44].

The Fourier transform infrared spectroscopy (FTIR) spectrum of avidin and Av–Au NCs was illustrated in Additional file 1: Fig. S1B. The broad peak at 3340 cm^−1^ was attributed to stretching of the amino group (NH_2_) [45]. And the peaks observed at 2965 cm^−1^ and 1155 cm^−1^ were responsible for C-H and C-N stretching frequencies respectively in both curves [46]. The sharp peak at 1638 cm^−1^ was assigned to the C = O stretching vibration, and the amide (II) peak at 1541 cm^−1^ originated from peptide bonds in avidin [47]. The results showed that the avidin had the characteristic functional groups of carboxyl (COOH) and amino (NH_2_). When avidin was used to serve as a protecting ligand for the Au NCs formation, the characteristic peaks of Av–Au NCs from the corresponding FTIR spectrum (red line) exhibited a slight decrease in comparison to characteristic peaks of avidin. The reason could be due to conformational changes that were happening after cluster formation.

### MALDI-TOF mass spectra and XPS spectra of Av–Au NCs

X-ray photoelectron spectroscopy (XPS) was performed to detect the chemical state of gold in Av–Au NCs that emit pink fluorescence. According to the literature, the gold core of Au_8_ NCs is completely composed of Au (0) [20], whereas that of Au_25_ NCs is composed of Au (0) and Au (I) [38]. Thus, the Av–Au NCs that emit pink fluorescence should contain both Au (0) and Au (I). As shown in Additional file 1: Fig. S2A, the XPS spectrum of Au 4f spectra was fitted. Two distinct doublet Au 4f_7/2_ peaks (green and purple curves) were centered at 84.46 eV and 86.14 eV, slightly higher than Au (0) and Au (I) of BSA-stabilized Au NCs [15], which might be attributed to the fact that the synthesized Av–Au NCs contain smaller clusters. This result indicates that Au (III) was reduced to Au (0) and Au (I) by avidin and grew to form Au NCs.

MALDI-TOF mass spectrometry (MS) was conducted to reveal the molecular weight of avidin and Av–Au NCs to determine the number of gold atoms in Av–Au NCs. As shown in Additional file 1: Fig. S2B, the molecular weight of avidin was 67,307 (gray line); after synthesizing Av–Au NCs, this peak shifted to 68,830 (red line), suggesting that there are eight Au atoms in the Av–Au NCs, which might explain the blue fluorescence of Av–Au NCs. However, we found that the intensity values of the mass spectrum peak were low, and there was no mass spectrum peak of the large gold cores. This might be caused by the following reasons: (1) the template concentration of the synthesized Av–Au NCs in the experiments was much lower than that of other Prot-Au NCs; (2) avidin was degraded during synthesis and storage; and (3) the number of large Av–Au NCs was small.

### Optimum concentration of HAuCl_4_ for the synthesis of Av–Au NCs

**Fig. 2:**
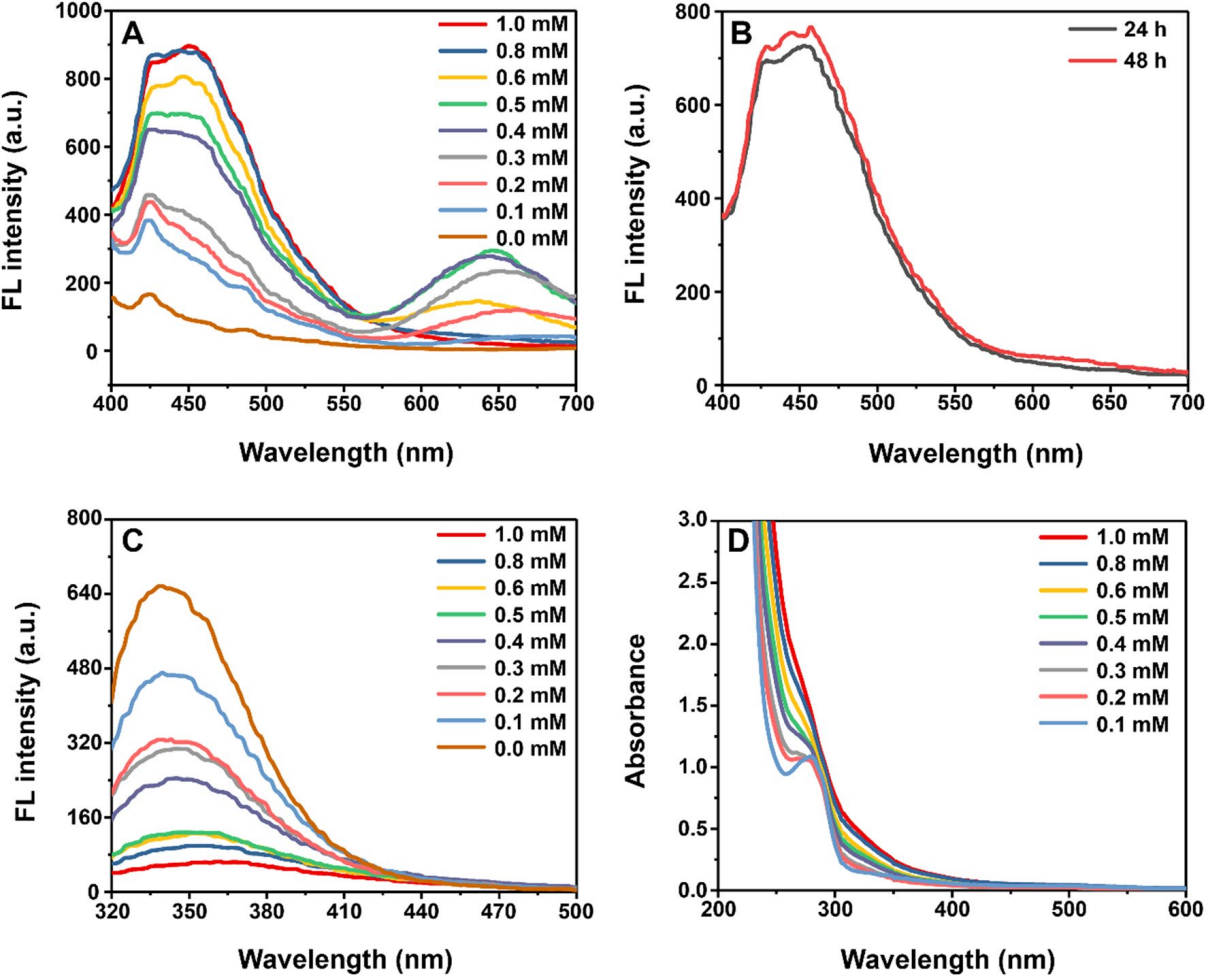
Optimum concentration of HAuCl_4_ for the synthesis of Av–Au NCs. **A** Fluorescence emission spectra for Av–Au NCs synthesized from different concentrations of HAuCl_4_ with excitation at 374 nm. Slit widths of excitation: 10 nm, slit widths of emission: 10 nm, and voltage: 700 V. **B** Fluorescence emission curves of 24 h and 48 h Av–Au NCs synthesized from 1.0 mM HAuCl_4_ as a raw material. Slit widths of excitation: 10 nm, slit widths of emission: 5 nm, and voltage: 700 V. **C** Fluorescence emission spectra for Av–Au NCs synthesized from different concentrations of HAuCl_4_ with excitation at 295 nm. Slit widths of excitation: 5 nm, slit widths of emission: 5 nm, and voltage: 650 V. **D** Optical absorption spectra of Av–Au NCs synthesized from different concentrations of HAuCl_4_. Different concentrations of HAuCl_4_ were allowed to react in a 38 °C water bath for 10 h

Next, different concentrations of HAuCl_4_ were selected for synthesis to explore the relationship between the fluorescence emission spectrum of Av–Au NCs and the concentration of synthetic raw materials. While the concentration of avidin was fixed at 2 mg/mL, the concentration of HAuCl_4_ was adjusted from 0.1 mM to 1.0 mM, and then the fluorescence intensity changes of the synthesized Av–Au NCs were observed. Figure 2A shows that an increase in the concentration of HAuCl_4_ increased the fluorescence intensity of Av–Au NCs until 0.8 mM of HAuCl_4_, after which the fluorescence intensity did not increase further. The red fluorescence only appeared at the concentration of 0.2 mM to 0.6 mM of HAuCl_4_. To verify that the red fluorescence of the Av–Au NCs synthesized with a fixed concentration of avidin was related to a specific concentration range of HAuCl_4_, we extended the incubation time to 24 h and 48 h for the 1.0 mM HAuCl_4_ concentration group. Figure 2B demonstrated that the Av–Au NCs incubated for 24 h and 48 h had no red fluorescence, indicating that the red fluorescence was related to the concentration of HAuCl_4_.

Considering that the synthesis process of Au NCs might affect the structure of avidin, we monitored the changes in the local protein environment by monitoring the fluorescence strength of tryptophan (Trp) excited at 295 nm. Trp, as an intrinsic fluorophore group, shows decreased emission intensity or is accompanied by a red-shift in the emission maximum owing to partial expansion or solvent exposure of proteins, and it is frequently used to monitor changes in the local structure of proteins [41]. It could be concluded from Fig. 2C that the higher the concentration of HAuCl_4_, the lower the fluorescence intensity at 350 nm, indicating that the concentration of HAuCl_4_ affects the structure of avidin.

In addition, it was found that the Av–Au NC composites synthesized with different concentrations of HAuCl_4_ did not have an absorption peak at 520 nm through ultraviolet–visible (UV–Vis) absorption spectroscopy (Fig. 2D), indicating that no gold nanoparticles appeared in the synthesized composites. Therefore, we selected an HAuCl_4_ concentration of 0.4 mM to synthesize Av–Au NCs with dual fluorescence emission wavelengths to study the relationship between fluorescence emission changes and incubation time.

### Optimum incubation time for the synthesis of Av–Au NCs

**Fig. 3:**
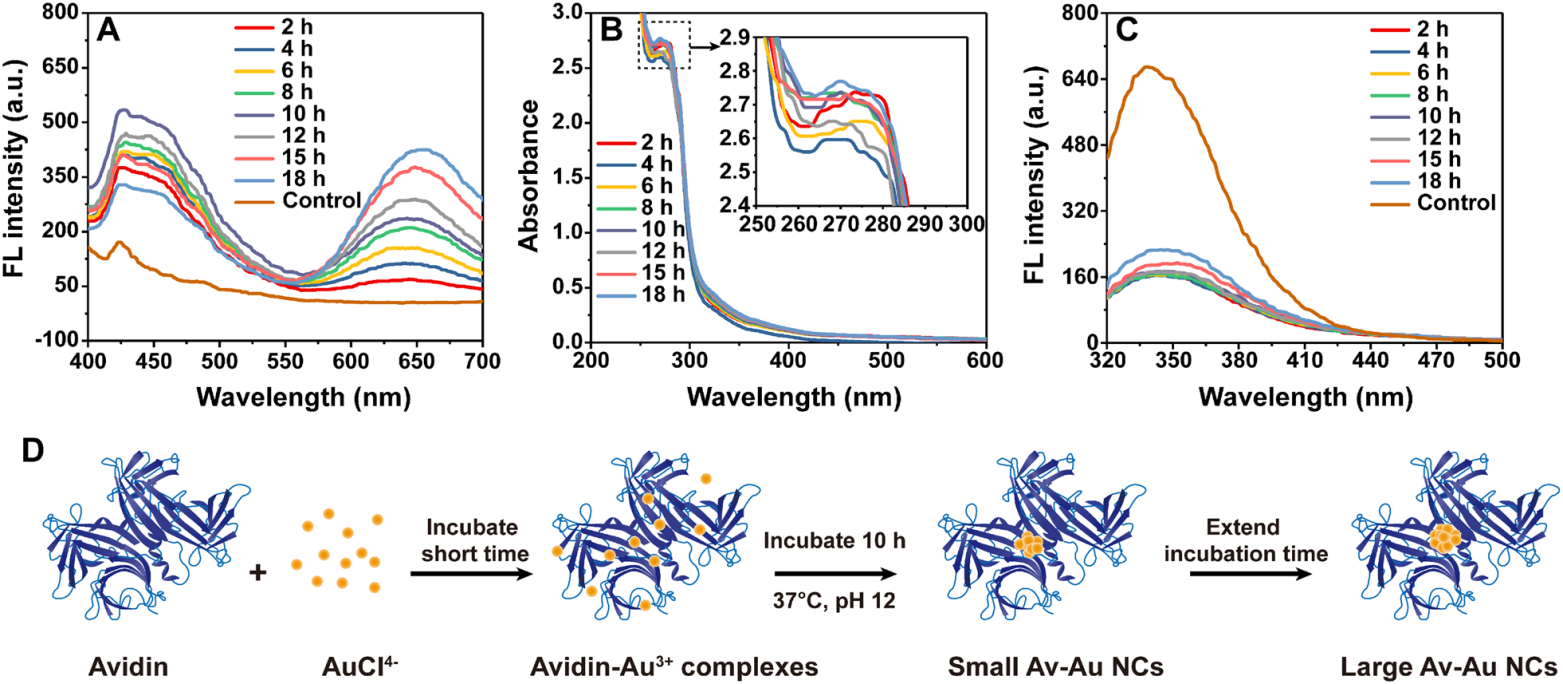
Optimum incubation time for the synthesis of Av–Au NCs. **A** Time-dependent fluorescence spectra obtained from the synthesis of Av–Au NCs with excitation at 374 nm. Slit widths of excitation: 10 nm, slit widths of emission: 10 nm, and voltage: 700 V. **B** Time-dependent absorption spectra obtained from the synthesis of Av–Au NCs. **C** Time-dependent fluorescence spectra obtained from the synthesis of Av–Au NCs with excitation at 295 nm. Slit widths of excitation: 5 nm, slit widths of emission: 5 nm, and voltage: 650 V. **D** Growth mechanism for the synthesis of Av–Au NCs with blue fluorescence and red fluorescence

To observe the changes in the fluorescent emission peaks of Av–Au NC compounds over time, the emission spectra of the reaction solution of avidin (200 µL, 2 mg/mL) with HAuCl_4_ (200 µL, 0.4 mM) at 38 ℃ were detected at different incubation times. Figure 3A shows the time-dependent fluorescence spectra of Av–Au NCs. During the first 10 h of incubation, the intensity of the fluorescence peak at ~ 449 nm gradually increased, and a new band centered at approximately 651 nm increased prominently with the increase in incubation time. This phenomenon was attributed to the conversion of the avidin-Au^3+^ complexes to small Au NCs and large Au NCs. From the 12th to 18th hour, the emission peak at ~ 449 nm gradually decreased, while the emission peak at ~ 651 nm continued to increase; this conversion might be due to the gradual transition from small Au NCs to large Au NCs.

Besides, the reaction system incubated for 8 h of Fig. 3A was placed at 4 ℃ for more than 3 months to observe the change of the fluorescence curve. It was detected that the fluorescence intensity of the Av–Au NCs increased significantly at 651 nm after being placed at 4 ℃ for more than 3 months, but the fluorescence intensity at 449 nm increased not significantly (Additional file 1: Fig. S3), which might be due to the slow and continuous synthesis of large Au clusters at low temperature. The experimental results also showed that the Av–Au NCs exhibited high photostability under storage conditions.

The time-dependent evolution of Av–Au NC compounds was also monitored by UV–Vis spectroscopy (Fig. 3B). The UV–Vis absorption spectra of the Av–Au NC compounds were similar at different incubation times, and there was no absorption peak around 520 nm, which indicated that gold nanoparticles were not synthesized during the long-term reaction.

The conformational changes of avidin with incubation time were tracked by the changes in Trp emission profile. As shown in Fig. 3C, the Trp emission curve revealed a decrease in Trp emission intensity compared to the native avidin protein for all the Av–Au NC systems studied. However, the fluorescence intensity of Trp was gradually restored from the 12th to the 18th h, indicating that the structure of avidin was affected by the formation of large Au clusters.

We propose the following as a possible growth mechanism of Av–Au NCs (Fig. 3D). First, cysteine, histidine, and other residues of avidin bind to Au^3+^ through coordination [33, 48]. As the reaction proceeds, these bound Au^3+^ ions are reduced to form mainly small Au NCs as well as a small proportion of large Au NCs by tryptophan/tyrosine residues in alkaline solution in the first 10 h. Finally, some of the small Au NCs are reduced to form large Au NCs due to more Au^3+^ reduction, thereby decreasing the blue fluorescence and increasing the red fluorescence.

### Verification of the binding ability of Av–Au NCs to biotin

**Fig. 4:**
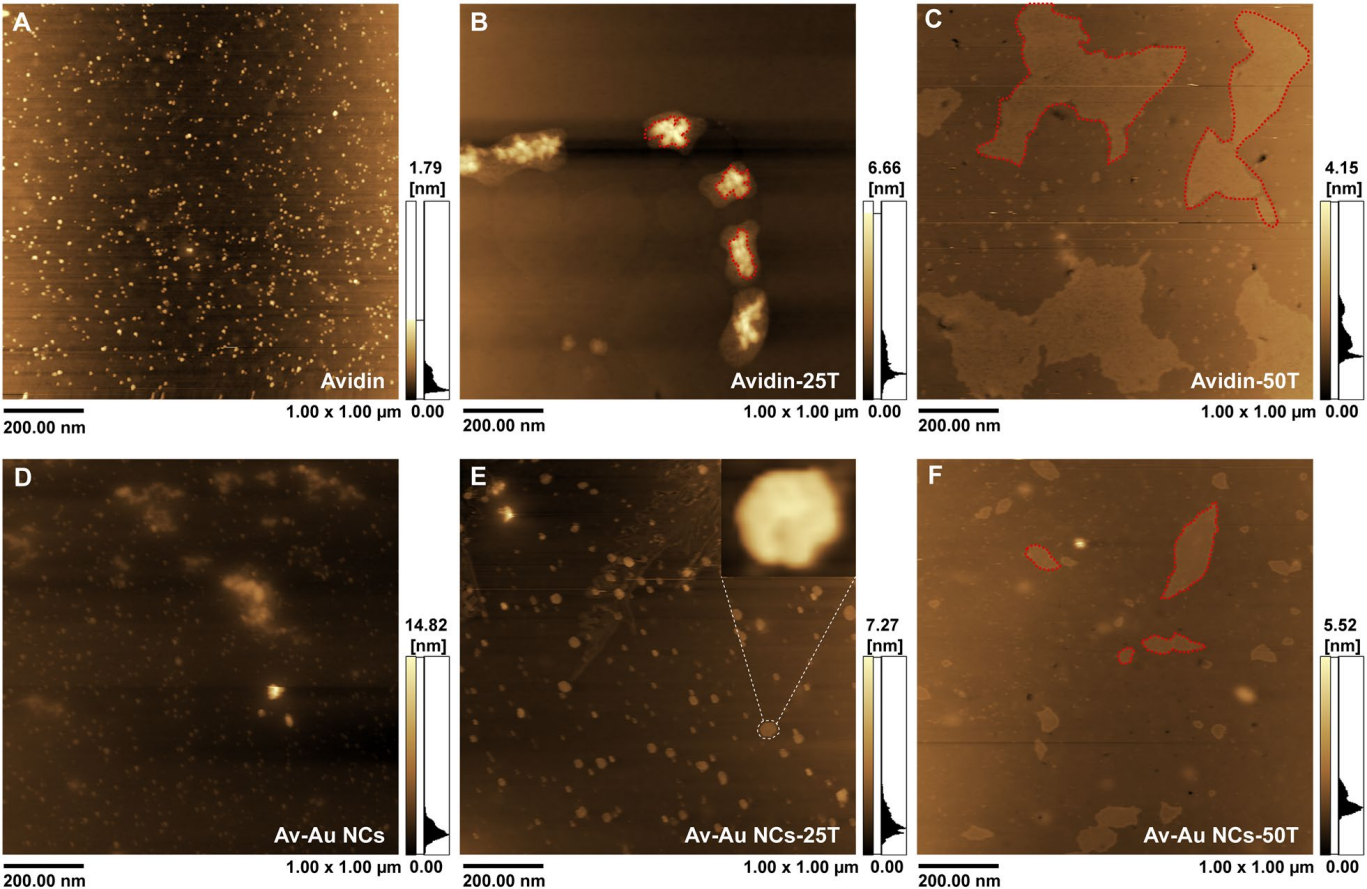
Verification of the binding ability of Av–Au NCs to biotin. AFM images of avidin (**A**), Avidin-25 T (**B**), Avidin-50 T (**C**), Av–Au NCs (**D**), Av–Au NCs-25 T (**E**), and Av–Au NCs-50 T (**F**) (scale: 200 nm). The inset of part (**E**) is an amplified AFM image of an Av–Au NCs-25 T complex

**Fig. 5:**
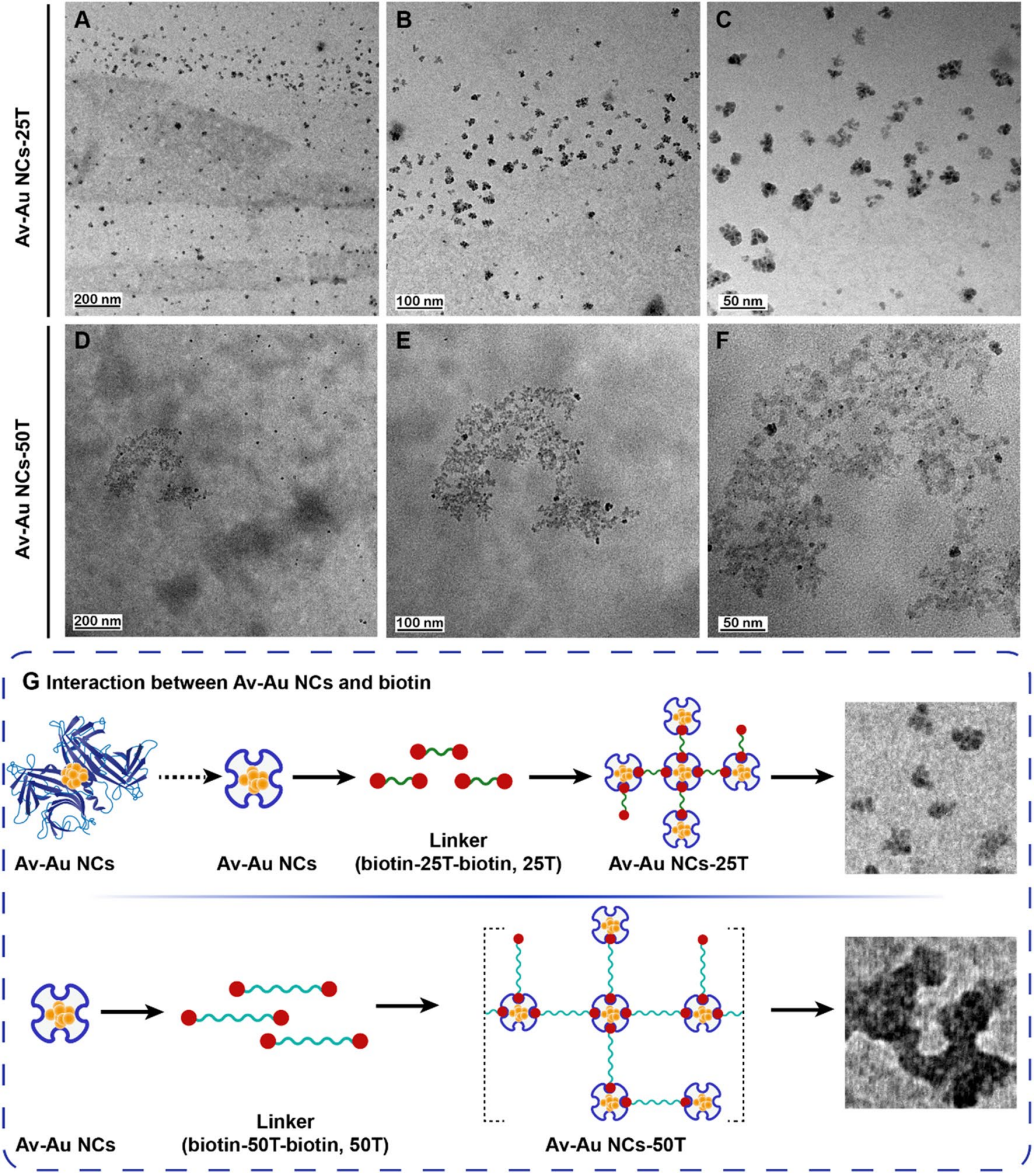
Interaction between Av–Au NCs and the DNA strands modified with biotin at both ends was confirmed with high-resolution transmission electron microscopy (HRTEM). HRTEM images with different magnifications of Av–Au NCs-25 T formed by the reaction of Av–Au NCs and 25 T (**A**, **B**, **C**), and Av–Au NCs-50 T formed by the reaction of Av–Au NCs and 50 T (**D**, **E**, **F**). **G** Diagram of the interaction between Av–Au NCs and DNA strands with biotin modified at both ends (up: 25 T, down: 50 T)

To verify whether Av–Au NCs have the same ability to bind to biotin molecules, we designed DNA strands modified with biotin at both ends for incubation with Av–Au NCs. When Av–Au NCs bonded with biotin, large polymers were formed, and the agglomeration of Av–Au NCs could be observed under atomic force microscopy (AFM) (Fig. 4) and HRTEM (Fig. 5). As shown in Fig. 4A, we chose pure avidin protein as the control group to verify the binding ability of Av–Au NCs to biotin. Figure 4A and D demonstrate that the avidin and the synthesized Av–Au NCs were uniformly distributed. Following incubation of avidin and Av–Au NCs with oligonucleotides consisting of 25 thymine nucleotides modified with biotin at both ends (25 T), the large plum-shaped structure (Avidin-25 T; the structure surrounded by the red dotted line; Fig. 4B) and the small plum-shaped structure (Av–Au NCs-25 T; Fig. 4E) were formed. This indicated that Av–Au NCs could bind to biotin, but the binding ability was decreased compared to pure avidin, which might be due to the structural changes of avidin during the synthesis of Au NCs. The formation of the plum-like cluster rather than sheet-like polymers may be due to the steric hindrance caused by the short 25 T.

Next, the length of the nucleic acid chains was extended to 50 bases (50 T) to achieve a better aggregation effect and determine whether larger clusters could be formed. Figure 4C shows that avidin could form large flake avidin aggregates (Avidin-50 T; the structure surrounded by the red dotted line) after incubation with 50 T. Simultaneously, Fig. 4F shows that incubation of Av–Au NCs with 50 T led to the formation of small flake aggregates of Av–Au NCs (Av–Au NCs-50 T; the structure surrounded by the red dotted line). The results demonstrate that the length of the nucleic acid chain affects the clustering effect of Av–Au NCs.

In addition, the interaction between Av–Au NCs and the DNA strands modified with biotin at both ends was further observed by HRTEM (Fig. 5). As shown in the high-definition electron micrographs of Fig. 5A–C, small plum-like Au NCs were formed following the incubation of Av–Au NCs with 25 T. Figure 5D–F show the interaction of Av–Au NCs with 50 T to form large gold clusters, which was consistent with the experimental results of Fig. 4. The synthetic Av–Au NCs retained the ability to bind to biotin, and they could form large clusters by interacting with the nucleic acid strands of the biotin modified at both ends of the appropriate length. Therefore, the synthetic Av–Au NCs have good potential to be used in the field of biosensing. To better understand this effect, the binding effect of Av–Au NCs with 25 T (up) and 50 T (down) is shown in Fig. 5G.

In addition, dynamic light scattering (DLS) experiments were used to observe the size of the polymers formed by Av–Au NCs after long term incubation with different linker chains. 5 µL of 50 T or 25 T (100 µM) was added to 95 µL of Av–Au NCs solutions, respectively. The mixed solution was then incubated at 4 ℃ for 3 days. After that, the size distribution of the polymers formed in solution could be detected by DLS (Malvern Zetasizer Nano ZS90). As shown in Additional file 1: Fig. S4, the polymers formed by Av–Au NCs-50 T were larger than that of Av–Au NCs-25 T. Moreover, the size of the formed polymers of both groups were larger compared with the results of HRTEM and AFM, which might be due to prolonging the incubation time of Av–Au NCs with different linker chains to 3 days. Therefore, the results demonstrate the size stability of the Av–Au NCs binding to different linker chains.

### Av–Au NCs for sensing of target DNA

Avidin functionalized Au NCs that possess dual functions, namely, fluorescence and the ability to bind to biotin, could be designed for biomarker detection. The construction of a target DNA detection system based on the Av–Au NCs-biotin signal amplification system is shown in Scheme 1. Initially, the MBs were synthesized and the capture probe (Cp) was modified on the surface of the MBs to form Cp@MB; the synthesis and modification processes of the MBs are shown in Additional file 1: Fig. S5. TEM image analysis of the synthesized MBs confirmed the spherical and uniform nature of the magnetite core and revealed that the diameter of MBs was 343.5 ± 28.2 nm (Additional file 1: Fig. S6). We also determined whether Cp was bound to MBs using UV–Vis absorption spectroscopy (Additional file 1: Fig. S7).

**Fig. 6:**
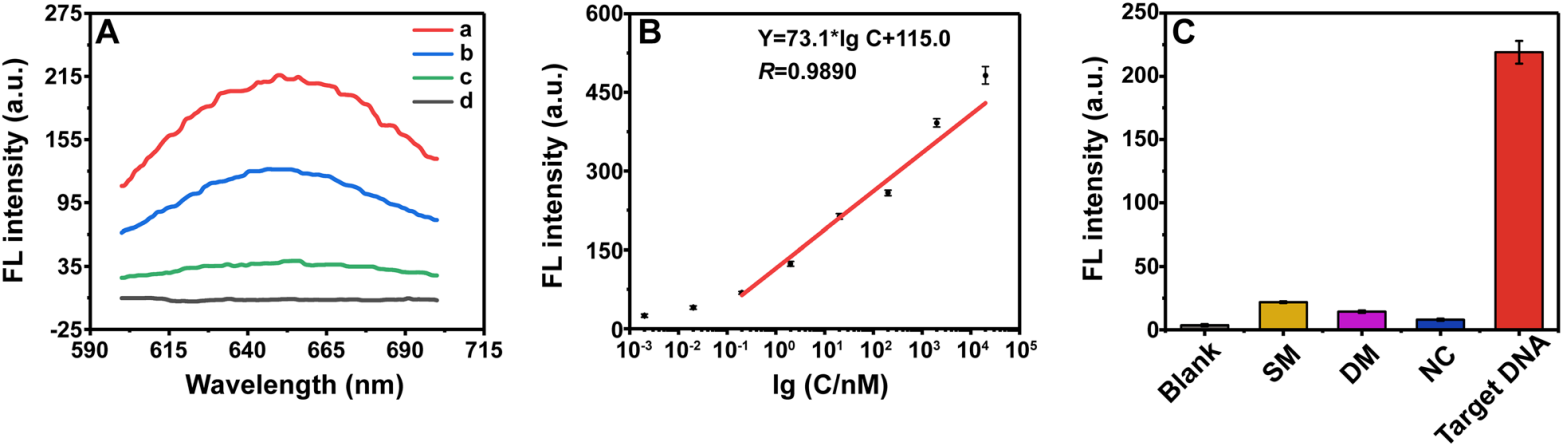
Av–Au NCs for sensing of target DNA. **A** The fluorescence intensity curves of the feasibility experience. Curve a, b, c and d represent addition of Av–Au NCs-50 T, Av–Au NCs-25 T, Av–Au NCs and PBS, respectively. The concentration of the target DNA was 20 nM. Slit widths of excitation: 10 nm, slit widths of emission: 10 nm, and voltage: 700 V. **B** Calibration plots of the fluorescence signals vs. the logarithm of target DNA concentrations from 0.2 nM to 2 × 10^4^ nM. Error bar represents the standard deviation (*n* = 3). **C** Fluorescence response signals for the specificity of target DNA (20 nM) detection against blank, SM (20 nM), DM (20 nM), and NC (20 nM). Error bar represents the standard deviation (*n* = 3).

To explore the feasibility of the functional Av–Au NCs-50 T system-based fluorescent biosensor, we chose an oligonucleotide DNA probe as the model target for experiments, and we designed Cp DNA and helper DNA that were complementary to the target DNA (Additional file 1: Table S1). The interaction between the three was proved by 12% native PAGE (Additional file 1: Fig. S8). It was found that the emission spectrum of the silicon dioxide layer on the surface of the MBs under an excitation light of 374 nm overlapped with the blue spectrum of the Av–Au NCs; therefore, we chose the red fluorescence of the Av–Au NCs as the detection signal in the subsequent experiments. The results of the feasibility experiences are shown in Fig. 6A. Compared to the PBS control groups (curve d), the experimental groups of Av–Au NCs-50 T (curve a) exhibited a clear fluorescence signal at 651 nm after 50 µL of 20 nM target DNA was added, which clearly demonstrated the feasibility of the purpose strategy. And the fluorescence intensity of the experimental groups of Av–Au NCs-50 T (curve a) was higher than that of Av–Au NCs-25 T (curve b) and Av–Au NCs (curve c). Thus, Av–Au NCs-50 T was selected for follow-up experiments.

Then, to investigate the detection sensitivity of the biosensing method, different concentrations of target DNA were added for verification. By increasing the concentration of target DNA, the fluorescence spectra showed a gradual increase in fluorescence at 651 nm. The resulting linear regression equation was Y = 73.1 × Ig C + 115.0 (*R* = 0.9890), where Y and C denote the fluorescent signal and the concentration of target DNA, respectively (Fig. 6B). The linear range was 0.2 nmol/mL to 2 × 10^4^ nmol/mL, and the limit of detection (LOD) for the target DNA was calculated to be 0.043 nM according to the 3σ rule. As the ability of avidin to bind biotin was impaired during Av–Au NCs synthesis, the sensitivity of nucleic acid detection was lower than that of some other reported methods based on magnetic separation (Additional file 1: Table S2). We could either further increase the fluorescence intensity of Av–Au NCs or reduce the impaired binding ability of Av–Au NCs to biotin to realize the detection of low-abundance samples.

Three DNA mismatch DNA samples, including single-base mismatch (SM), double-base mismatch (DM), and non-complementary DNA (NC) were used to evaluate the selectivity of the biosensor. As shown in Fig. 6C, the fluorescence signal intensity caused by target DNA was significantly higher than that of other groups, indicating that the constructed biosensor based on Av–Au NCs displayed good selectivity for the target DNA. The above experiments show that the novel enzyme-free biosensor based on the Av–Au NCs-biotin system has a promising application prospect for DNA detection.

## Conclusions

In summary, Av–Au NCs were synthesized for the first time by a water-bath method. Av–Au NCs with dual emission wavelengths were synthesized by adjusting the ratio of HAuCl_4_ to avidin and the incubation time. More importantly, the generated Av–Au NCs retained the native ability of avidin to bind to biotin, which could be used to construct biosensors for target DNA detection. Subsequently, a highly sensitive and specific biosensor based on the Av–Au NCs-biotin signal amplification system was constructed for detecting target DNA. This biosensing method avoids the introduction of biological enzymes and the tedious modification process required for fluorescent probes. However, for samples with lower abundance, the Av–Au NCs-biotin signal amplification system requires further optimization. The parameters for optimization mainly include three aspects: improving the fluorescence intensity of Av–Au NCs; maintaining the ability of Av–Au NCs to bind to biotin as much as possible; and optimizing the length of the linker chain. The Av–Au NCs-biotin signal amplification system was found to be capable of detecting DNA in liquid samples, and we believe that, in the future, immunofluorescence (IF) and fluorescence in situ hybridization (FISH) based on Av–Au NCs-biotin system could be used to achieve specific and sensitive detection of other target substances. Thus, Av–Au NCs have significant application potential in the fields of biosensing.

## Materials and methods

### Materials and reagents

Phosphate buffer saline (PBS; pH 7.2–7.4) was supplied by Thermo Fisher Scientific (Wilmington, USA). GelRed nucleic acid stain was acquired from SenBeiJia Biological Technology Co., Ltd. (Nanjing, China). NHydroxysuccinimide (NHS) and N-(3-(dimethylamino) propyl)-N′-ethylcarbodiimide hydrochloride (EDC) were purchased from Alfa Aesar (Ward Hill, MA, USA). Chloroauric acid (HAuCl_4_), sodium hydroxide (NaOH), FeCl_3_·6H_2_O, ethylene glycol (EG), tetraethylorthosilicate (TEOS), 3-aminopropyltriethoxysilane (APTES), and 28% ammonium hydroxide (NH_3_·H_2_O) were obtained from Sinopharm Chemical Reagent Co., (Shanghai, China). Dialysis membrane (3.5 kDa), sodium acetate (NaAC), sodium citrate (Na_3_Cit), avidin (activity ≥ 12 U/mg), and all of the HPLC-purified oligonucleotides listed in Additional file 1: Table S1 were prepared and purified by Sangon Biotech Co., Ltd. (Shanghai, China). All of the reagents and chemicals used were of analytical reagent grade. Ultrapure water was prepared by a Millipore Milli-Q gradient ultrapure water system (Millipore, MA).

### Apparatus

The fluorescence signal was recorded using a Cary Eclipse fluorescence spectrophotometer (Agilent Technologies, Palo Alto, CA). Polyacrylamide gel electrophoresis (PAGE) was performed on a DYY-6C electrophoresis analyzer (Liuyi Instrument Company, China) and imaged on a Bio-rad ChemDoc XRS (Bio-Rad, USA). UV–Vis absorption spectra were obtained using a UV-2550 spectrophotometer (Shimadzu, Kyoto, Japan). A high-resolution transmission electron microscope (FEI Tecnai G2 F20, USA) and an atomic force microscope (Shimadzu, Japan) were used to characterize the size, morphology, and thickness of the Av–Au NCs and Av–Au NCs-biotin supramolecular complexation. A transmission electron microscope (Hitachi, Tokyo, Japan) was used to characterize the morphologies of the synthetic MBs. The absolute QY measurements and the PL quantum yields of Av–Au NCs were obtained by an Edinburgh FLS-1000 fluorescence spectrophotometer. The molecular structures of Av–Au NCs were observed on a Fourier transform infrared (FTIR) spectroscopy system (Nicolet 670, USA). The FTIR spectral data of Av–Au NCs and avidin was processed using Omnic software (Thermo Scientific, USA). X-ray photoelectron spectroscopy (XPS) was performed to explore the electronic structures of the Av–Au NCs on a Thermo Scientific K-Alpha XPS instrument. The XPS spectra of the Au 4f core levels were deconvoluted using XPSPEAK software (Version 4.0). The sp3 C1s peak was used as a reference for binding energy calibration. The molecular weights of avidin and Av–Au NCs were analyzed with MALDI-TOF mass spectrometry on a Bruker Daltonics Autoflex III TOF/TOF system.

### Synthesis and purification of Av–Au NCs

In a typical synthesis protocol, aqueous HAuCl_4_ solution (200 µL, 0.4 mM) was added to avidin solution (200 µL, 2 mg/mL) under vigorous stirring for 3 min. Next, 1 M NaOH was added to adjust the pH to 12, and the mixture was incubated at 38 °C for 24 h to complete the reaction. The as-prepared Av–Au NC compound was filtered with a 3.5 kDa dialysis membrane to adjust the pH of the Av–Au NC solution to neutral. Finally, the size, shape, and dispersion state of the Av–Au NCs were characterized using AFM, HRTEM, and UV–Vis spectrophotometry (wavelength range from 250 to 600 nm), and the Av–Au NCs were stored at 4 °C until further use.

### Preparation of the Av–Au NCs-25 T and Av–Au NCs-50 T composites

The dialyzed Av–Au NCs (95 µL) were mixed with 50 T or 20 T (5 µL, 100 µM). To form the Av–Au NCs-25 T and Av–Au NCs-50 T composites, each mixture was incubated for 1 h at room temperature before verification by HRTEM or AFM.

### Native polyacrylamide gel electrophoresis (native-PAGE)

The base pairing of DNA strands was evaluated by 12% native-PAGE in 1 × TBE buffer (Tris-boric acid, EDTA, pH 8.3) at 110 V constant voltage for 50 min at 4 °C. Then, the gels were stained with GelRed nucleic acid dye for 20 min and imaged by a gel image system.

### Fabrication of the Av–Au NCs-biotin supramolecular complexation biosensor

Initially, a specified concentration of target DNA was added to 10 µL of Cp@MB suspension, and the mixture was incubated for 30 min at room temperature. Subsequently, 2 µL of 1 µM helper DNA was added to the mixture and incubated at room temperature for 30 min. After that, the unlinked nucleic acid strands were removed by magnetic separation. Then, 100 µL Av–Au NCs and 2 µL 100 µM 50 T nucleic acid strands were added to form a polymer through the interaction between biotin and Av–Au NCs. Next, MBs were magnetically extracted to remove unreacted components. Finally, the above magnetic bead complex was resuspended in 100 µL PBS solution for fluorescence detection. A washing step is necessary after each incubation step to remove the excess DNA strands or Av–Au NCs. The fluorescence emission spectrum was recorded in the wavelength range of 600–700 nm, with an excitation wavelength of 374 nm (slit widths of excitation: 10 nm, slit widths of emission: 10 nm, and voltage: 700 V).

## Supplementary Information

**Additional file 1: Table S1.** Sequence of oligonucleotides employed in the experiments. The colors show related sequences (through complementarity or similarity). **Figure S1.** (A) XRD spectrum of Av-Au NCs. (B) The FTIR spectrum of Av-Au NCs (red line) and avidin (grey line). Figure S2. (A) XPS spectrum of Av-Au NCs. (B) MALDI-TOF mass spectra of avidin and Av-Au NCs. **Figure S3.** Fluorescence curves of Av-Au NCs after synthesis (b) and after storage at 4 ℃ for more than 3 months (a) with excitation at 374 nm. Slit widths of excitation: 10 nm, slit widths of emission: 10 nm, and voltage: 700 V. **Figure S4.** The size distribution of the polymer formed by Av-Au NCs-50T/25T after incubation of Av-Au NCs with 50T or 25T for 3 days at 4 ℃. **Figure S5.** Synthetic strategies for capture probe@magnetic beads (Cp@MB). (A) synthesis of magnetic beads. (B) The decoration of the magnetic beads. **Figure S6.** (A) TEM image and (B) size distribution of the magnetic beads. **Figure S7.** UV-Vis absorption of the capture probe in the supernatant. (a) Before and (b) after cross-linking of the capture probe with MBs. **Figure S8.** 12% native PAGE analysis of the complementary base pairing between capture DNA, target DNA, and helper DNA: 50 bp DNA ladder marker (lane M), 1 µM capture DNA (lane 1), 1 µM target DNA (lane 2), 1 µM helper DNA (lane 3), the mixture of 1 µM capture DNA, 1 µM target DNA and 1 µM helper DNA (lane 4). **Table S2.** Comparison the performance of detection of the proposed method with some reported methods based on magnetic separation.

## Data Availability

All data analyzed during this study are included in this published article.

## Abbreviations

Prot-Au NCs: Protein-stabilized gold nanoclusters
Av–Au NCs: Avidin-stabilized gold nanoclusters
Au NCs: Gold nanoclusters
BSA: Bovine serum albumin
HRP: Horseradish peroxidase
MBs: Magnetic beads
PLQY: Photoluminescence quantum yield
HRTEM: High-resolution transmission electron microscopy
MS: Mass spectrometry
Trp: Tryptophan
25 T: 25 Thymine nucleotides modified with biotin at both ends
DLS: Dynamic light scattering
LOD: Limit of detection
SM: Single-base mismatch
DM: Double-base mismatch
NC: Non-complementary DNA
IF: Immunofluorescence
FISH: Fluorescence in situ hybridization
PBS: Phosphate buffer saline
NHS: NHydroxysuccinimide
EDC: N-(3-(dimethylamino) propyl)-N′-ethylcarbodiimide hydrochloride
HAuCl_4_·4H_2O_: Chloroauric acid hydrated
NaOH: Sodium hydroxide
EG: Ethylene glycol
TEOS: Tetraethylorthosilicate
APTES: 3-Aminopropyltriethoxysilane
NH_3_·H_2_O: Ammonium hydroxide
NaAC: Sodium acetate
Na_3_Cit: Sodium citrate
PAGE: Polyacrylamide gel electrophoresis
UV–Vis: Ultraviolet–visible
AFM: Atomic force microscopy
TEM: Transmission electron microscopy
XRD: X-ray diffraction
FTIR: Fourier transform infrared spectroscopy
XPS: X-ray photoelectron spectroscopy
RT: Room temperature
Native-PAGE: Native polyacrylamide gel electrophoresis
Cp: Capture probe
MBs: Magnetic beads
Cp@MB: Capture probe@magnetic bead complexes
