# Supplementary material for: Fabrication of avidin-stabilized gold nanoclusters with dual emissions and their application in biosensing
Source: J Nanobiotechnology. 2022 Jun 27;20:306. doi: 10.1186/s12951-022-01512-8 (PMC9235210; doi:10.1186/s12951-022-01512-8)
Supplement: Supplementary file 1 — Additional file 1: Table S1. Sequence of oligonucleotides employed in the experiments. The colors show related sequences (through complementarity or similarity). Figure S1. (A) XRD spectrum of Av-Au NCs. (B) The FTIR spectrum of Av-Au NCs (red line) and avidin (grey line). Figure S2. (A) XPS spectrum of Av-Au NCs. (B) MALDI-TOF mass spectra of avidin and Av-Au NCs. Figure S3. Fluorescence curves of Av-Au NCs after synthesis (b) and after storage at 4 ℃ for more than 3 months (a) with excitation at 374 nm. Slit widths of excitation: 10 nm, slit widths of emission: 10 nm, and voltage: 700 V. Figure S4. The size distribution of the polymer formed by Av-Au NCs-50T/25T after incubation of Av-Au NCs with 50T or 25T for 3 days at 4 ℃. Figure S5. Synthetic strategies for capture probe@magnetic beads (Cp@MB). (A) synthesis of magnetic beads. (B) The decoration of the magnetic beads. Figure S6. (A) TEM image and (B) size distribution of the magnetic beads. Figure S7. UV-Vis absorption of the capture probe in the supernatant. (a) Before and (b) after cross-linking of the capture probe with MBs. Figure S8. 12% native PAGE analysis of the complementary base pairing between capture DNA, target DNA, and helper DNA: 50 bp DNA ladder marker (lane M), 1 µM capture DNA (lane 1), 1 µM target DNA (lane 2), 1 µM helper DNA (lane 3), the mixture of 1 µM capture DNA, 1 µM target DNA and 1 µM helper DNA (lane 4). Table S2. Comparison the performance of detection of the proposed method with some reported methods based on magnetic separation. [file 12951_2022_1512_MOESM1_ESM.docx]

**Supporting information**

**Fabrication of avidin-stabilized gold nanoclusters with dual emissions and their application in biosensing**

**Zhenrong Tang^1†^, Fengjiao Chen^2†^, Dan Wang^3^, Dongmei Xiong^4^, Shaoying Yan^5^, Hua Tang^3*^ and Shengchun Liu^1*^**

1. *Department of Endocrine and Breast Surgery, The First Affiliated Hospital of Chongqing Medical University, Chongqing 400042, China.*
2. *Guangshan County People’s Hospital, Xinyang, Henan 465450, China*
3. *Key Laboratory of Molecular Biology for Infectious Diseases (Ministry of Education), Institute for Viral Hepatitis, Department of Infectious Diseases, The Second Affiliated Hospital, Chongqing Medical University, Chongqing 400016, China.*
4. *Nursing School of Chongqing Medical and Pharmaceutical College, Chongqing 401331, China.*
5. *Department of Clinical Laboratory, The First Affiliated Hospital of Nanchang University, Nanchang, Jiangxi 330006, China*

**^†^**Zhenrong Tang and Fengjiao Chen contributed equally to this work.

******* Corresponding author: Shengchun Liu *or* Hua Tang

1 Yi Xue Yuan Road, Chongqing, 400016, China

Tel: +86 23 68486780. Fax: +86 23 68486780.

E-mail: liushengchun1968@163.com *or* [tanghua86162003@cqmu.edu.cn](mailto:tanghua86162003@cqmu.edu.cn)

**Table S1** Sequence of oligonucleotides employed in the experiments. The colors show related sequences (through complementarity or similarity).

| Name | Sequence (5'-3') |
| --- | --- |
| Target DNA | TGCCTTGTAAGAGCGACGTAGGTGAATGAG |
| Capture probe | COOH-TTTTTTTTTTCTCATTCACCTACG |
| Helper DNA | TCGCTCTTACAAGGCATTTTTTTTTTTTTTTTTTTTTTTTTTTTTTTTTT-Biotin |
| 50T DNA | Biotin-TTTTTTTTTTTTTTTTTTTTTTTTTTTTTTTTTTTTTTTTTTTTTTTTTT-Biotin |
| 25T DNA | Biotin-TTTTTTTTTTTTTTTTTTTTTTTTT -Biotin |
| SM | TGCCTTGTAAGAGCGATGTAGGTGAATGAG |
| DM | TGCCTTGTAAGAGCGATGTAGGTGAATTAG |
| NC | TCGACCTGGGCAGGGTTCGCAGATCCTGCGACGTA |

SM: Signal-base-mismatched; DM: double-base-mismatched; NC: non-complementary oligonucleotide.

**XRD and FTIR studies of Av-Au NCs.**


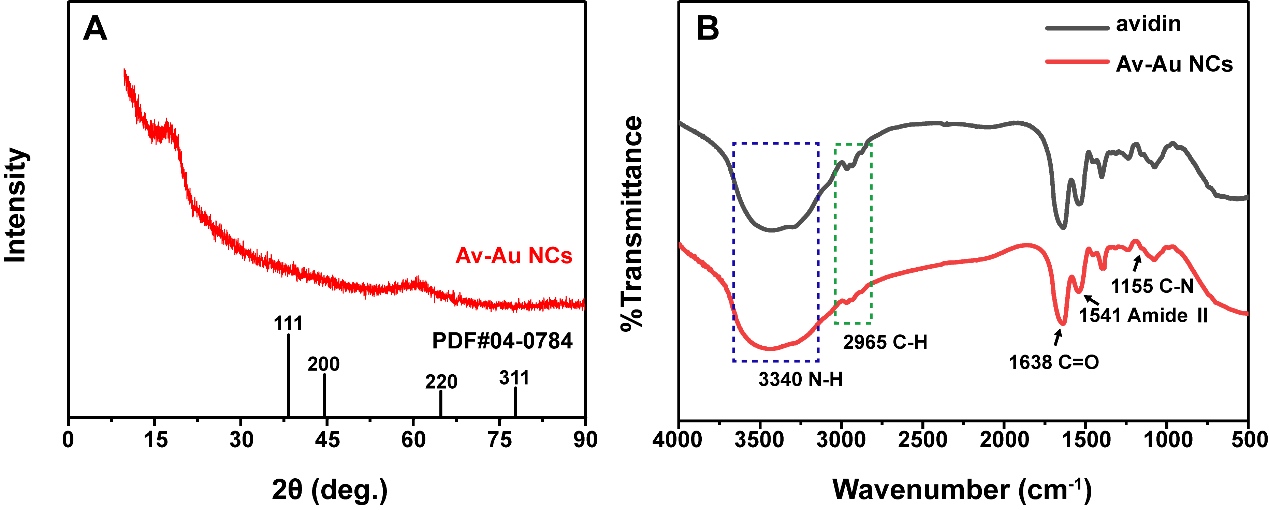


**Fig. S1** (**A**) XRD spectrum of Av-Au NCs. (**B**) The FTIR spectrum of Av-Au NCs (red line) and avidin (grey line).

**MALDI-TOF mass spectra and XPS spectra of Av-Au NCs**


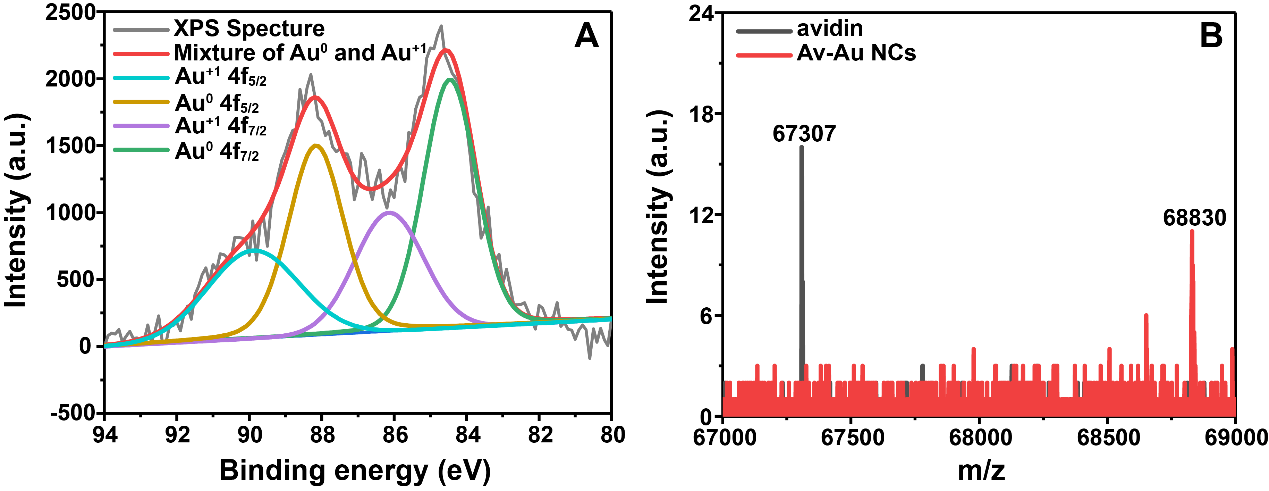


**Fig. S2** (**A**) XPS spectrum of Av-Au NCs. (**B**) MALDI-TOF mass spectra of avidin and Av-Au NCs.

**Photostability of Av-Au NCs at 4 °C**


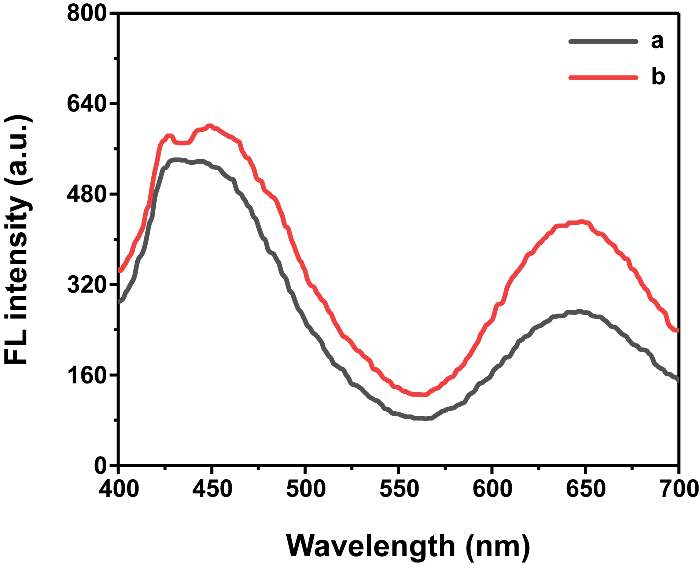


**Fig. S3** Fluorescence curves of Av-Au NCs after synthesis (b) and after storage at 4 ℃ for more than 3 months (a) with excitation at 374 nm. Slit widths of excitation: 10 nm, slit widths of emission: 10 nm, and voltage: 700 V.

**DLS experiments**


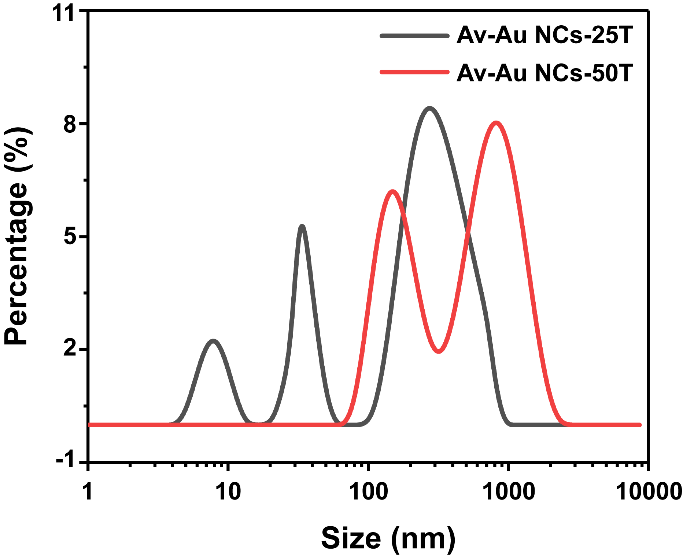


**Fig. S4** The size distribution of the polymer formed by Av-Au NCs-50T/25T after incubation of Av-Au NCs with 50T or 25T for 3 days at 4 ℃.

**Synthesis of magnetic beads (MBs)**

MBs were prepared according to method outlined in a previous report [1], with a slight modification. The preparation of MB was performed in a three-step process (Fig. S5A) as follows: (Ⅰ) Synthesis of Fe_3_O_4_ NPs. Initially, 0.67 g FeCl_3_·6H_2_O was dissolved in 12.5 mL ethylene glycol (EG), followed by the addition of 1.35 g anhydrous sodium acetate (NaAC) and 0.5 g anhydrous sodium citrate (Na_3_Cit). This mixture was then mixed using ultrasound until fully dissolved, before decanting into a white Teflon-lined autoclave. After heating at 200°C for 6 h, the autoclave was cooled down to room temperature (RT) to obtain a black Fe_3_O_4_ NP suspension. The black product was washed several times with ethanol and dried at RT. (Ⅱ) Silicidation of Fe_3_O_4_ NPs. Subsequently, the 0.1 g Fe_3_O_4_ NPs were immersed into HCl (15 mL, 1 M) and stirred for 10 min. The partially dissolved Fe_3_O_4_ NPs were isolated by a magnet, washed with water, and re-suspended in 10 wt% aqueous sodium citrate (Na_3_Cit) followed by stirring for 30 min. The citrate complexed Fe_3_O_4_ NPs were magnetically separated. Then, a mixture of 80 mL ethanol, 20 mL of water, and 2 mL ammonia solution (28 wt%) was transferred into the citrate complexed Fe_3_O_4_ NPs, followed by 15 min ultrasonication treatment. Next, 1 mL TEOS (99%) was added drop wise to the resultant mixture followed by mechanical stirring (400 rpm) for 12 h at RT. The prepared Fe_3_O_4_@SiO_2_ nanoparticles were magnetically separated and washed repeatedly with ethanol. The Fe_3_O_4_@SiO_2_ NPs were immersed in HCl (4 N) for 10 min to remove bare iron oxide NPs. The separated particles were then washed with water and air dried at RT. (Ⅲ) Amination of Fe_3_O_4_ NPs@SiO_2_. First, 0.2 g of Fe_3_O_4_@SiO_2_ NPs was dissolved in 70 ml isopropanol, before adding 0.1 mL APTES with vigorous stirring for 6 h at 70°C in a round-bottomed flask. Finally, the brown powder was washed with water and dried at RT.

**Synthesis of the capture probe@ magnetic beads (Cp@MB) complex**

The preparation of Cp-magnetic bead conjugates was based largely on previous methods [2]. As shown in Fig. S5B, 40 mg NHS and 320 mg EDC were added to 0.65 mL well solution of 0.1 M MES buffer (pH 5.5) to activate the capture probe (1.5 μM). The resultant mixture was gently shaken at RT for 0.5 h. Then, 350 μL amino-modified magnetic beads (20 mg/mL) were transferred into the solution. Thereafter, aqueous NaOH solution was added to adjust the pH to 9, followed by gentle shaking at 4°C for 24 h to synthesize the Cp@MB complex. After that, the supernatant was separated magnetically with deionized water for three times to remove the unreacted capture probe. The resulting Cp@MB complex was re-dispersed in 1.4 mL PBS and stored at 4°C.


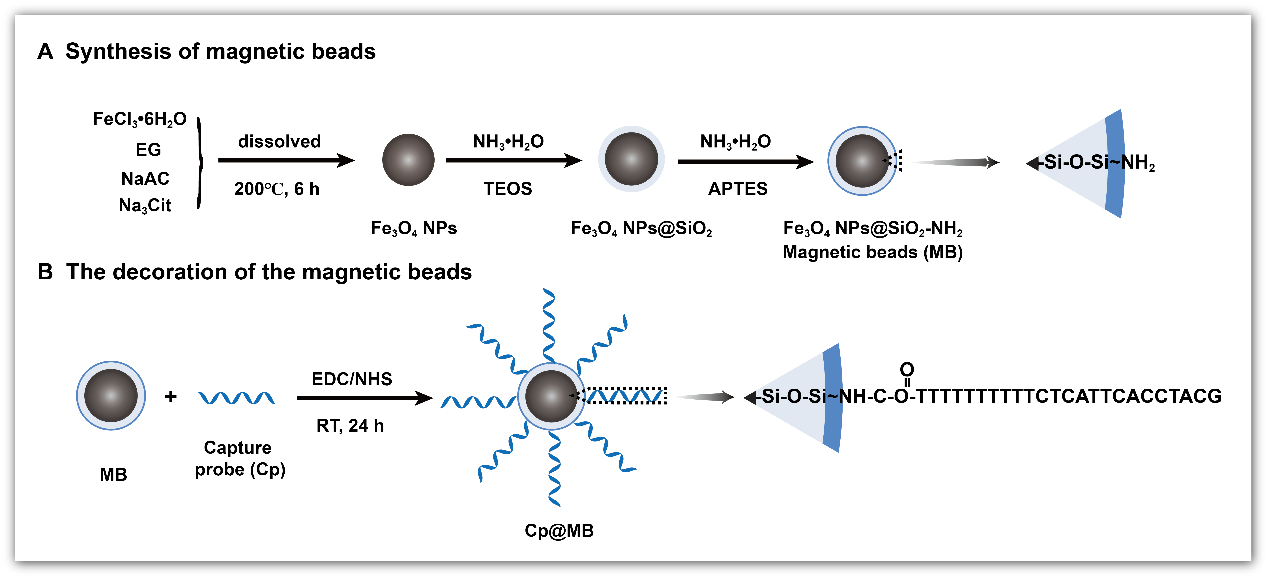


**Fig. S5** Synthetic strategies for capture probe@magnetic beads (Cp@MB). (**A**) synthesis of magnetic beads. (**B**) The decoration of the magnetic beads.

**Characterization of MBs**

The morphology and structure of MBs were characterized by TEM. As shown in Fig. S6A, the obtained magnetic beads were regular spheres with a diameter of approximately 343.5 nm (Fig. S6B).


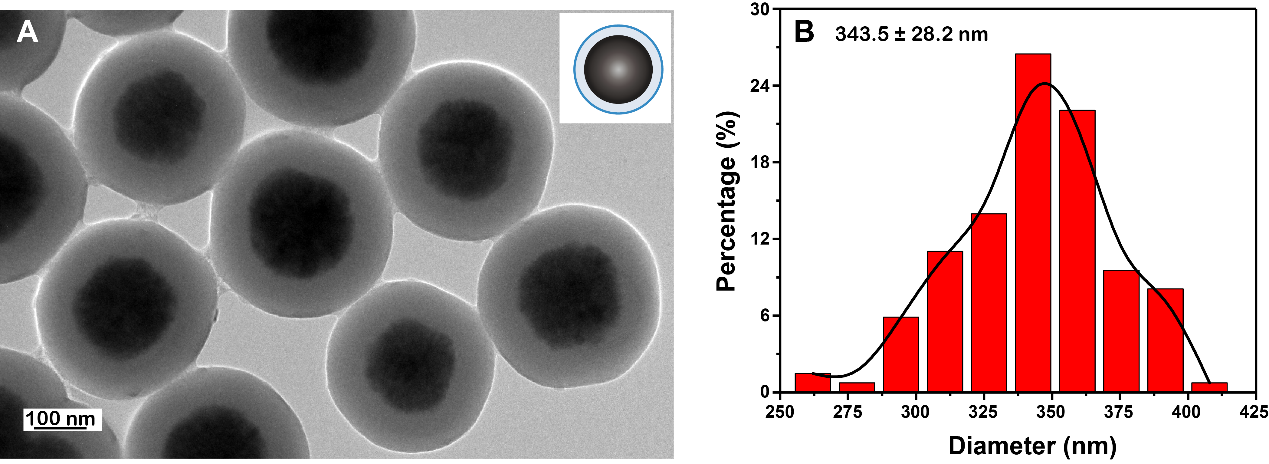


**Fig. S6** (**A**) TEM image and (**B**) size distribution of the magnetic beads.

**UV-Vis absorption characterization**

To verify whether the capture probe (Cp) was modified to MBs, we measured the absorption peak of the supernatant at 260 nm before and after Cp conjugation to MBs. As shown in Fig. S7, the absorbance was obviously decreased after the Cp reacted with MBs, indicating that the Cp was successfully bound to MBs.


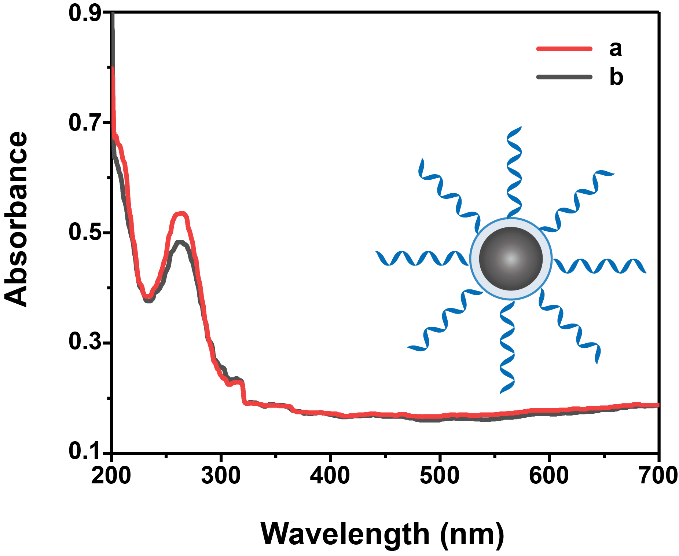


**Fig. S7** UV-Vis absorption of the capture probe in the supernatant. (a) Before and (b) after cross-linking of the capture probe with MBs.

**Characterization** **of the interaction of DNA strands**

First, 12% native PAGE was used to investigate whether the designed nucleic acid strand could be paired with complementary bases. As shown in Fig. S8, the migration speed of capture DNA (lane 1), target DNA (Lane 2), and helper DNA (lane 3) gradually decreased with the increase in the length of the single chain. The mixture of capture DNA, target DNA, and helper DNA could form a new stripe in lane 4, indicating that three DNA strands could form a sandwich structure.


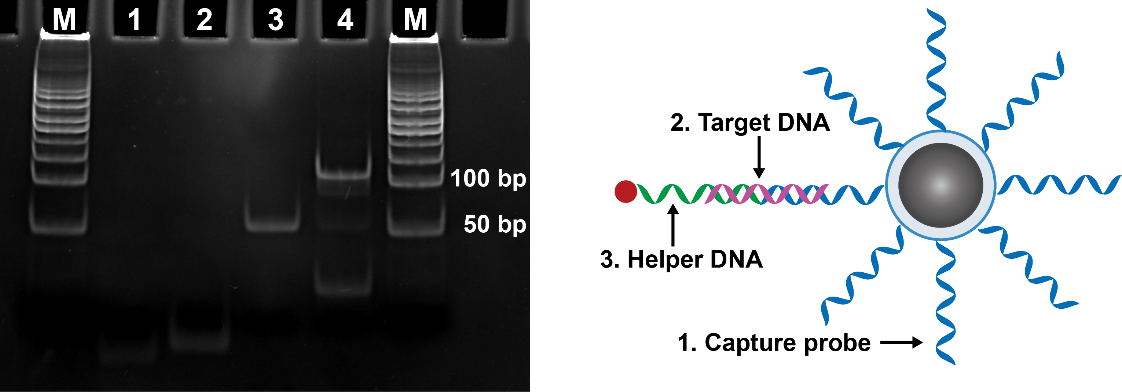


**Fig. S8** 12% native PAGE analysis of the complementary base pairing between capture DNA, target DNA, and helper DNA: 50 bp DNA ladder marker (lane M), 1 µM capture DNA (lane 1), 1 µM target DNA (lane 2), 1 µM helper DNA (lane 3), the mixture of 1 µM capture DNA, 1 µM target DNA and 1 µM helper DNA (lane 4).

**Table S2.** Comparison the performance of detection of the proposed method with some reported methods based on magnetic separation.

| Signal  amplification | Analytical  method | Linear  range (nM) | LOD  (nM) | Reference |
| --- | --- | --- | --- | --- |
| HCR/Pd-Au NRs | SERS | 10^-8^-10^-1^ | 3.116×10^-9^ | [3] |
| MNAzyme | ICP-MS | 5×10^-3^-2 | 1.15×10^-3^ | [4] |
| CRISPR-Cas12a | Colorimetry | 10^-4^-5 | 4.18×10^-5^ | [5] |
| DSN | SERS | 3.3×10^-7^-3.3×10^-3^ | 4.2×10^-8^ | [6] |
| Strep-HRP | Electrochemistry | 3-10^2^ | 0.91 | [7] |
| Av-Au NCs-biotin | Fluorescence | 0.2-2×10^4^ | 4.3×10^-2^ | This work |

HCR: Hybridization chain reaction; Pd-Au NRs: Pd-Au core-shell nanorods; MNAzyme: multicomponent nucleic acid enzyme; DSN: duplex-specific nuclease; Strep-HRP: streptavidin-horseradish peroxidase conjugate;

SERS: Surface enhanced Raman scattering; ICP-MS: inductively coupled plasma mass spectrometry.

**References**

1. Liu WB, Yang BJ, Wan LY, Wen L, Gao MZ. Synthesis of Magnetic Particles and Silica Coated Core-Shell Materials. Adv Mat Res. 2013;631-632**:**490-3.

2. Dong H, Chen H, Jiang J, Zhang H, Cai C, Shen Q. Highly Sensitive Electrochemical Detection of Tumor Exosomes Based on Aptamer Recognition-Induced Multi-DNA Release and Cyclic Enzymatic Amplification. Anal Chem. 2018;90**:**4507-13.

3. Cao X, Ge S, Zhou X, Mao Y, Sun Y, Lu W, et al. A dual-signal amplification strategy based on pump-free SERS microfluidic chip for rapid and ultrasensitive detection of non-small cell lung cancer-related circulating tumour DNA in mice serum. Biosens Bioelectron. 2022;205**:**114110.

4. Liu S, Wu J, He M, Chen B, Kang Q, Xu Y, et al. DNA Tetrahedron-Based MNAzyme for Sensitive Detection of microRNA with Elemental Tagging. ACS Appl Mater Interfaces. 2021;13**:**59076-84.

5. Gong S, Zhang S, Wang X, Li J, Pan W, Li N, et al. Strand Displacement Amplification Assisted CRISPR-Cas12a Strategy for Colorimetric Analysis of Viral Nucleic Acid. Anal Chem. 2021;93**:**15216-23.

6. Yao Y, Zhang H, Tian T, Liu Y, Zhu R, Ji J, et al. Iodide-modified Ag nanoparticles coupled with DSN-Assisted cycling amplification for label-free and ultrasensitive SERS detection of MicroRNA-21. Talanta. 2021;235**:**122728.

7. Povedano E, Ruiz-Valdepeñas Montiel V, Gamella M, Serafín V, Pedrero M, Moranova L, et al. A novel zinc finger protein-based amperometric biosensor for miRNA determination. Anal Bioanal Chem. 2020;412**:**5031-41.
